# Genetic Characterization and Pathogenesis of Avian Influenza Virus H7N3 Isolated from Spot-Billed Ducks in South Korea, Early 2019

**DOI:** 10.3390/v13050856

**Published:** 2021-05-07

**Authors:** Thuy-Tien Thi Trinh, Indira Tiwari, Kaliannan Durairaj, Bao Tuan Duong, Anh Thi Viet Nguyen, Hien Thi Tuong, Vui Thi Hoang, Duong Duc Than, SunJeong Nam, Seon-Ju Yeo, Hyun Park

**Affiliations:** 1 Zoonosis Research Center, Department of Infection Biology, School of Medicine, Wonkwang University, Iksan 570-749, Korea; trinhthithuytien.k56@hus.edu.vn (T.-T.T.T.); tiwariindira.micro@gmail.com (I.T.); kmdurairaj@gmail.com (K.D.); bao2dt@gmail.com (B.T.D.); nguyenthivietanh.k56@hus.edu.vn (A.T.V.N.); tuonghien23@gmail.com (H.T.T.); hoangvui169@gmail.com (V.T.H.); ducduong27189@gmail.com (D.D.T.); 2 Division of EcoScience, Ewha University, Seoul 03760, Korea; sjnam01@daum.net; 3 Department of Tropical Medicine and Parasitology, College of Medicine, Seoul National University, Seoul 03080, Korea

**Keywords:** avian influenza virus, H7N3, South Korea, spot-billed duck

## Abstract

Low-pathogenicity avian influenza viruses (LPAIV) introduced by migratory birds circulate in wild birds and can be transmitted to poultry. These viruses can mutate to become highly pathogenic avian influenza viruses causing severe disease and death in poultry. In March 2019, an H7N3 avian influenza virus—A/Spot-billed duck/South Korea/WKU2019-1/2019 (H7N3)—was isolated from spot-billed ducks in South Korea. This study aimed to evaluate the phylogenetic and mutational analysis of this isolate. Molecular analysis revealed that the genes for HA (hemagglutinin) and NA (neuraminidase) of this strain belonged to the Central Asian lineage, whereas genes for other internal proteins such as polymerase basic protein 1 (PB1), PB2, nucleoprotein, polymerase acidic protein, matrix protein, and non-structural protein belonged to that of the Korean lineage. In addition, a monobasic amino acid (PQIEPR/GLF) at the HA cleavage site, and the non-deletion of the stalk region in the NA gene indicated that this isolate was a typical LPAIV. Nucleotide sequence similarity analysis of HA revealed that the highest homology (99.51%) of this isolate is to that of A/common teal/Shanghai/CM1216/2017 (H7N7), and amino acid sequence of NA (99.48%) was closely related to that of A/teal/Egypt/MB-D-487OP/2016 (H7N3). An in vitro propagation of the A/Spot-billed duck/South Korea/WKU2019-1/2019 (H7N3) virus showed highest (7.38 Log_10_ TCID_50_/mL) virus titer at 60 h post-infection, and in experimental mouse lungs, the virus was detected at six days’ post-infection. Our study characterizes genetic mutations, as well as pathogenesis in both in vitro and in vivo model of a new Korea H7N3 viruses in 2019, carrying multiple potential mutations to become highly pathogenic and develop an ability to infect humans; thus, emphasizing the need for routine surveillance of avian influenza viruses in wild birds.

## 1. Introduction

Avian influenza is an infectious disease caused by the influenza A virus, which is widespread in migratory birds and mammals, including humans [1]. Waterfowl and wild birds are known to be the natural hosts and reservoirs of avian influenza viruses (AIVs). To date, AIVs have been identified on the basis of surface proteins, hemagglutinin (HA; H1–16 in birds and H17–18 in bats), and neuraminidase (NA; N1–9 in birds and N10–11 in bats) subtypes [2]. Additionally, they have been categorized into two groups based on their virulence: low-pathogenic AIVs (LPAIVs) and high-pathogenic AIVs (HPAIVs) [3].

Specifically, compared to the other 14 HA subtypes identified in birds, H5 and H7 have been mostly observed in wild avian species. Although the first report of a H7 subtype AIV, which was studied in northern Italy in 1878 by Perroncito, who labelled the disease caused by this virus as a “fowl plague” [4], long-term (1976–2012) surveillance reports from North American regions indicate that the H7 subtype HPAIVs mostly infect migratory birds [5,6]. Concomitantly, during that period, H7N1 and H7N3 species of the HPAIV H7 subtype caused outbreaks due to poultry/migratory birds [5], which spread to South/Latin American (Mexico and Canada), Europe (Italy), and South-Central Asian (Pakistan) regions [7,8].

Between 1995 and 2002, Aamir et al. reported that poultry, specifically chickens, were infected with HPAIV H7N3 in Pakistan; their report also clarified that this virus replicated poorly in mallard ducks [9]. In 2011, a total of four AIV H7N3 strains were isolated from domestic ducks in China, and has spread widely across the Asian regions [10]. In January 2017, H7N3 LPAIVs were isolated from ducks (*Anas platyrhynchos domesticus*) in Cambodia [11] and in March 2018, H7N3 AIVs from duck meat on a passenger flight from China to Japan—whose polybasic sequence was mutated to HPAIV H7N9—were identified as HPAIVs [12]. According to phylogenetic analysis in early 2011, Kim et al. reported that H7N3 AIVs have been co-circulating in South Korea [13]; although, detailed information on the pathogenicity of the H7N3 virus is still lacking.

The aim of this study is to evaluate the phylogenetic and mutational analysis of H7N3 AIVs from spot-billed ducks in South Korea, early 2019. Moreover, as there are currently no reports related to the pathogenetic analysis of H7N3 AIVs, to the best of our knowledge, we describe, for the first time in the literature, the complete pathogenesis of H7N3 AIVs by experimental infections in a mammalian model, in vitro and in vivo.

## 2. Materials and Methods

### 2.1. Sample Collection

A total of 1800 fresh samples of fecal droppings were collected during an annual surveillance from November 2018 to March 2019 in different regions of South Korea, stored at 2–8 °C, and shipped to the laboratory within a day for further analysis.

### 2.2. Isolation of Influenza Virus from Wildbird Feces

The wild bird fecal samples were pooled in groups of up to three and re-suspended in phosphate buffered saline (PBS) containing antibiotic solution (100 mg/µL of streptomycin) (Merck, St. Louis, MO, USA) followed by centrifugation at 3000 rpm for 10 min at 4 °C. To verify that the samples contained Influenza A viruses, reverse transcription polymerase chain reaction (RT-PCR) was performed to amplify the matrix gene, following the World Health Organization (WHO) guideline, using universal primers and probes [14]. The 0.1 mL filtered supernatants were each inoculated into the allatonic cavity of 9-day-old specific-pathogen-free (SPF) embryonated chicken eggs for virus isolation. Eggs were then incubated at 37 °C and candled daily for embryo viability assessment. At 3 days post-virus inoculation, eggs were incubated at 4 °C overnight. Following embryonic death or after 72 h post-infection (hpi), allantoic fluid was collected, and hemagglutinin assays of the allantoic fluid was determined with 0.5% packed chicken red blood cells, according to World Organization for Animal Health recommendations [15].

### 2.3. Host Identification

Host identification was confirmed by polymerase chain reaction (PCR) using a DNA barcode and a region, consisting of 751 base-pairs (bp), of the mitochondrial gene cytochrome *c* oxidase I (COI) as previously described [16]. The host of the new isolate was identified using a combination comparison between Barcode of Life Data System (BOLD; Biodiversity Institute of Ontario, University of Guelph, Guelph, ON, Canada) and Basic Local Alignment Search Tool for nucleotides (BLASTn; NCBI, National Institute of Health, Bethesda, MD, USA).

### 2.4. Reverse Transcription (RT)-PCR and Sequencing

Viral RNA was extracted directly from the allantoic fluid of embryonated chicken eggs (ECEs) using a NucleoSpin RNA kit (Macherey-Nagel, Düren, Germany) according to the manufacturer’s instructions. Briefly, virus-containing egg fluid was mixed with lysis buffer containing β-mercaptoethanol. Suspension viscosity was reduced by filtration in a Nucleospin filter, and RNA binding conditions were adjusted by adding ethanol. Solubilized RNA was bound to a Nucleospin RNA column membrane, and desalting was performed by the addition of a membrane desalting buffer. The DNA digestion step was omitted as rDNase treatment was noted to reduce RNA stability. Elution was performed directly after the desalted membrane was washed with wash buffers. The first strand cDNA was transcribed by a Superscript III first strand cDNA synthesis kit (Invitrogen, Carlsbad, CA, USA) using a universal primer for influenza A virus (Uni12: 5′-AGCRAAAGCAGG-3′) in a final volume of 20 µL as per the manufacturer’s protocol.

To evaluate the growth of the influenza virus and determine the subtype, conventional real-time RT-PCR for influenza A virus was performed using total RNA following World Health Organization guidelines [15].

### 2.5. Next Generation Sequencing (NGS) by Illumina Hiseq X Method

NGS sequencing was conducted by GnCBIO (Dae-Jeon, Korea) following the Hiseq X method, as previously reported [17,18]. Briefly, viral RNA was determined using an Ag-ilent’s BioAnalyzer 2100 and RNA 6000 pico chip ((For RIN measurement), (Agilent, Santa Clara, CA, USA), and the concentration was measured using a spectrophotometer. Ribosomal RNA was removed by using QIAGEN’s QIAseq FastSelect rRNA HMR Kit, followed by the cDNA synthesized using random primer and oligo dT primer. Then, the synthesized cDNA was amplified using the REPLI-g SensiPhi DNA polymerase enzyme in the kit to make a library. Library concentration was measured via the LightCycler qPCR (Roche, Penzberg, Upper Bavaria, Germany), and library size was verified using Ailent’s TapeStation D5000 ScreenTape system (Agilent, Santa Clara, CA, USA). For cluster generation, the library was loaded into a flow cell where fragments were captured on a lawn of surface-bound oligos complementary to the library adapters. Each fragment was then amplified into distinct, clonal clusters through bridge amplification. When cluster generation was complete, the templates were ready for sequencing. Sequencing data was converted into raw data for analysis.

Raw sequence reads were quality trimmed using “trim galore” (q = 20) and non-influenza virus read was removed using Deconseq (iden = 60). Python script was used as a tool to adjust the amount of data, up to 600,000 reads. Meanwhile a database of only segments four (HA), six (NA), and eight (NS1) from the influenza virus from NCBI was created to provide and align to those of the reference using Gsmapper (iden = 80, mL = 40). The open reading frame (ORF) was observed using the obtained consensus and adopted a result with an ORF similar to the reference. As the ORF length differed from the reference, sequence error was corrected using Proof Read as previously described [19].

### 2.6. Molecular Characterization and Phylogenetic Analysis

Nucleotide blast analysis was used to identify relevant viral genes, and reference sequences were downloaded from the National Center for Biotechnology Information database (NCBI https://www.ncbi.nlm.nih.gov/ (accessed on: 1 October 2020)) and the Global Initiative on Sharing All Influenza Data (GISAID https://www.gisaid.org/ (accessed on: 1 October 2020)). The data were merged, the duplicated sequences were deduced, and finally phylogenetic tree analyses were conducted using MEGA6.0 software (Molecular Evolutionary Genetics analysis version 6.0, Pennsylvania State University, PA, USA). Phylogenetic trees of all eight full length gene segments (*PB2*, *PB1*, *PA*, *HA*, *NP*, *NA*, *M,* and *NS*) of the H7N3 (WKU2019-1) isolate were generated by applying the neighbor-joining method with Kimura’s two-parameter distance model and 1000 bootstrap replicates. The HA and NA gene tree included sequences with high homology from the NCBI database, the H7N3 subtype reported from South Korea (complete genome), and a representative H7 HPAIV strain. The tree for the other six internal segments (*PB2*, *PB1*, *PA*, *NP*, *M*, and *NS*) included influenza virus isolate with nucleotide homologies to respective segments from different geographic regions [20,21].

### 2.7. Determination of 50% Tissue Culture Infectious Dose (TCID_50_) and 50% Egg Infectious Dose (EID_50_)

The enzyme-linked immunosorbent assay (ELISA) was used to measure the TCID_50_ (50% tissue culture infectious dose) titers as previously reported [14,22]. Briefly, Madin-Darby canine kidney (MDCK) cells (ATCC, Manassasa, VA, USA) were grown on flat-bottom 96-well plates at 37 °C with 5% CO_2_. Twenty-four hours after plating, cells with 80% confluence were washed with 1× phosphate-buffered saline (PBS) and inoculated with serial 10-fold dilutions of virus in media containing 1 µg/mL of L-1-tosylamide-2-phenylethyl chloromethyl ketonetreated trypsin (TPCK-trypsin). Virus-infected cells were incubated at 37 °C, 5% CO_2_ for 3 days and the TCID_50_ titers were determined via the Reed and Muench method [23]. For the determination of EID_50_, the chorioallantoic membrane (CAM) cavities of 10-day-old SPF embryonated chicken eggs were inoculated with 100 μL serial 10-fold dilutions of the viruses, five eggs for each dilution. The eggs were then incubated at 37 °C for 3 days. Allantoic fluid was harvested and tested via HA assays [24] and EID_50_ calculation of viruses was performed using the Reed and Muench method [23].

### 2.8. Viral Growth Kinetics in MDCK Cells

The growth kinetics of virus isolates were evaluated in in vitro. MDCK cells were inoculated with the viruses at a multiplicity of infection (MOI) of 0.01 in DMEM (Dulbecco’s Modified Eagle Medium) medium containing 1% antibiotic and 1 μg/mL TPCK-trypsin. Supernatants were collected at 12, 24, 36, 48, 60, 72, and 84 hpi. ELISA were performed with anti-influenza nucleoprotein (PAN antibody) to detect infected cells and the virus titer (TCID_50_) of each supernatant was determined [23].

### 2.9. Animal Experiment

The pathogenic potential of the new isolate was then determined in mammals; six-week-old female BALB/c mice purchased from Orient (Seongnam, Korea) (*n* = 11 mice’s/group), which were intranasally inoculated by 10^5^ EID_50_/mL of each viruses (i.e., H7N3 (WKU2019-1), and two control groups infected with A/California/04/2009 (H1N1) (CA/04/09) (pdm09) or A/mallard/Korea/KNUGPH12/2011 (H7N7) or mock infected as normal control group). Mice were anesthetized by 1% isoflurane following manufacturer’s instructions (Hana Pharmacy, Hwasung, Korea) according to the guidelines of Vertebrate Animal Research, University of Iowa [25]. The body weight and survival rate of mice were observed for 15 days. Following which, the mice were euthanized, and lungs (*n* = 3 mice/group) were collected at days 3, 6, and 15 post-infection (dpi). The lung tissue was homogenized and the TCID_50_ was determined to test the viral titers of homogenate supernatant [26]. For histopathology, the lungs of three mice were collected at 3, 6, and 15 dpi and stored in 10% formalin/saline at 4 °C until further use. Hematoxylin and eosin (H&E) were used for histopathological examination of paraffin-embedded lung tissues mounted on glass slides. All sections were observed using a light microscope (magnification × 100). This study was approved by the Animal Ethics Committee of the Wonkwang University (WKU19-64), 19 December 2019, and all methods were carried out in accordance with relevant guidelines and regulations.

### 2.10. Statistics

The mean, standard deviation (SD), and Student’s *t*-test were conducted using GraphPad Prism Software Version 5.0 (La Jolla, CA, USA). Results were presented as the mean ± SD. A value of *p* < 0.05 was considered significant.

## 3. Results

### 3.1. Genome Characterization of H7N3 (WKU2019-1) Isolate

Samples were isolated from feces of waterfowls in South Korea on 15 March 2019. RNA sample from feces were amplified for HA and NA using universal primer, and the PCR product was ligated to vector plasmid. Five different representative colonies were selected for HA/NA amplification and ligated sequence was sent for sequencing. The result showed positive for HA7 and NA3 subtype that initially confirmed H7N3 (Supplementary Figure S1). Further RNA isolated from feces along with allatonic fluid was directly sent for NGS. The detailed NGS analysis information are provided in Tables S1 and S2. Hence, our sample A/Spot-billed duck/South Korea/WKU2019-1/2019-1 (H7N3) is designated as H7N3 (WKU2019-1). The genome sequence information of the isolate was deposited in GenBank from accession numbers MT845654.1 to MT845661.1. The GenBank accession numbers of the eight gene segments and the highest nucleotide identities from the GenBank database are shown in Table 1, with the sequence identities from 97.85 to 100% when compared with our WKU2019-1 (H7N3) virus isolates.

Surface gene HA, matrix protein (M), and polymerase basic protein (PB)1 were closely related to A/common teal/Shanghai/CM1216/2017 (H7N7), which originated from China, while NA was closely related to A/teal/Egypt/MB-D-487OP/2016 (H7N3) with nucleotide identities of 99.51%, 99.90%, 99.56%, and 99.48%, respectively. PB2 was closely related to A/Duck/Hubei/HF5/2017 (H7N8), which originated from China, with a nucleotide identity of 99.17%. Similarly, the gene for polymerase acidic protein (PA) was closely related to A/wild duck/South Korea/KNU18-114/2018 (H7N7), was originated from Korea, with a nucleotide similarity of 99.54%. The gene for nucleoprotein (NP) was closely related to A/teal/Egypt/MB-D-487OP/2016 (H7N3) and A/teal/Egypt/MB-D-698OP/2016 (H7N3), which seemed to be originated from Egypt, while that for non-structural protein (NS) was closely related to A/wild goose/dongting lake/121/2018 (H6N2), from China, with nucleotide similarities of 99.66% and 99.65%, respectively.

### 3.2. Hypothesis for Reassortment Event for Each Gene Segment

**Figure 1:**
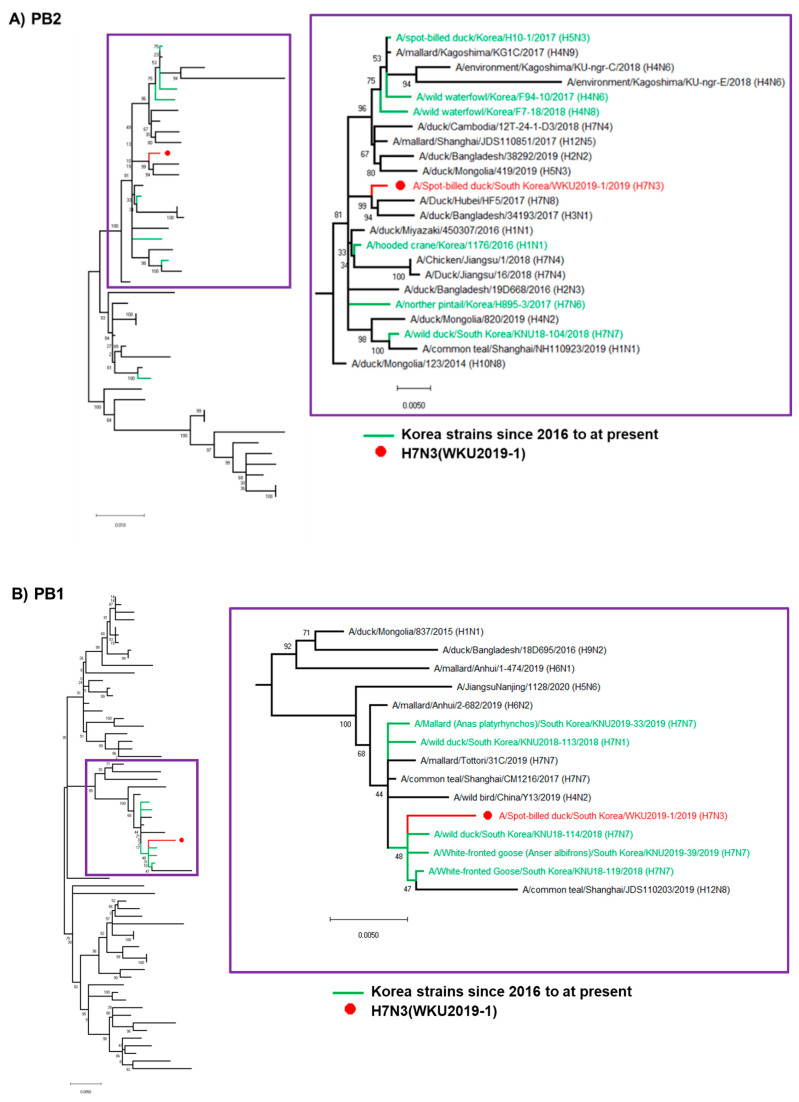

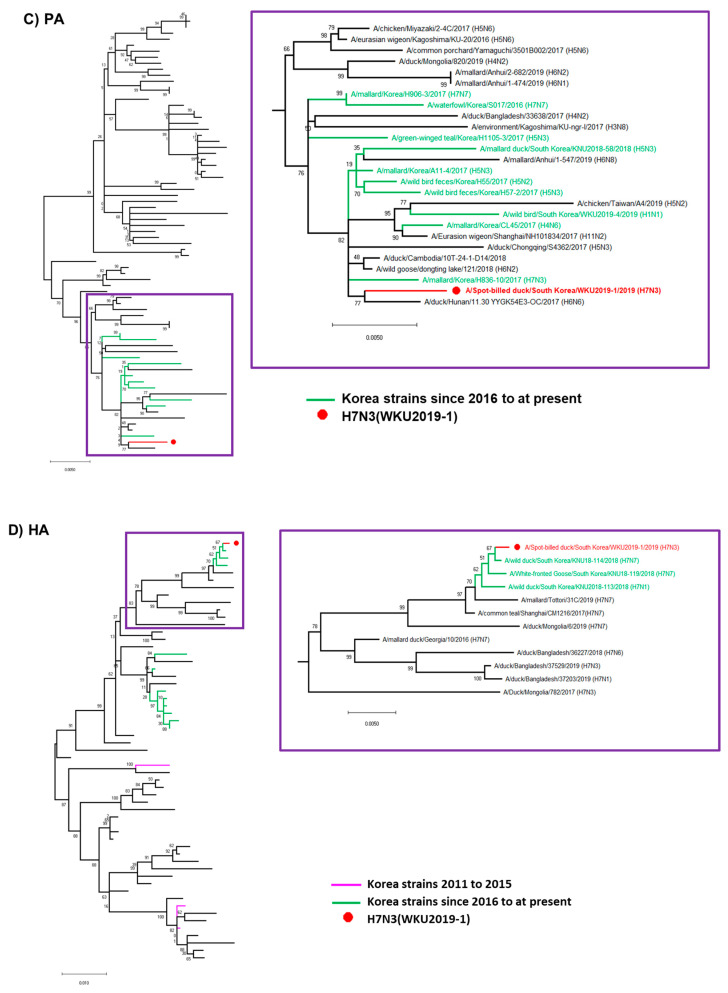

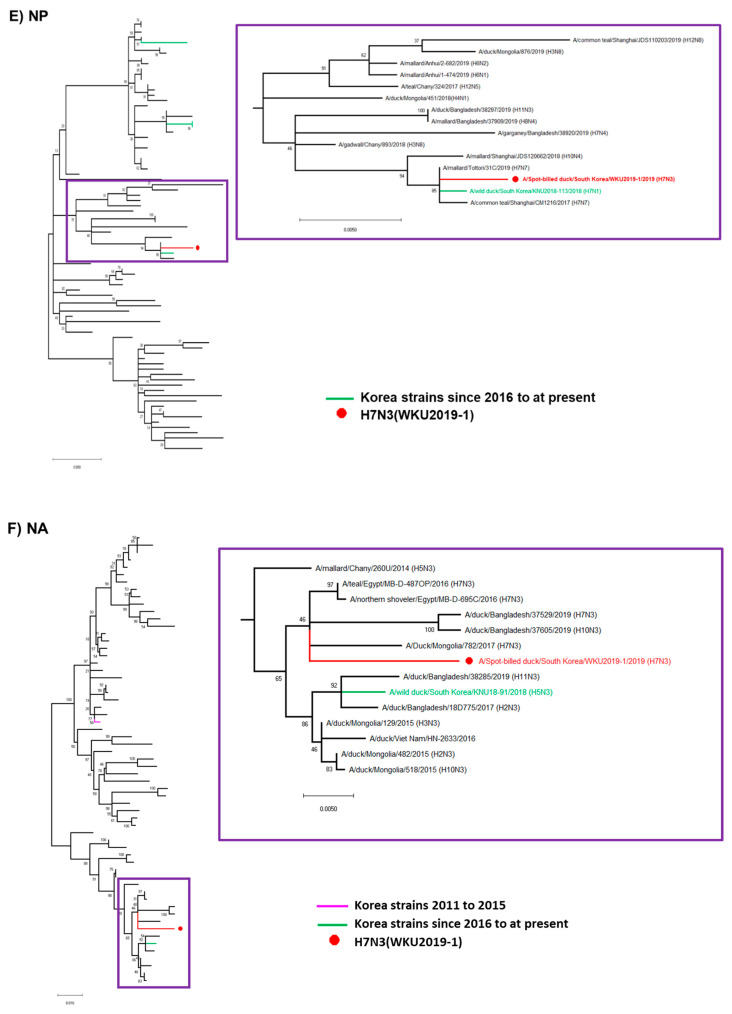

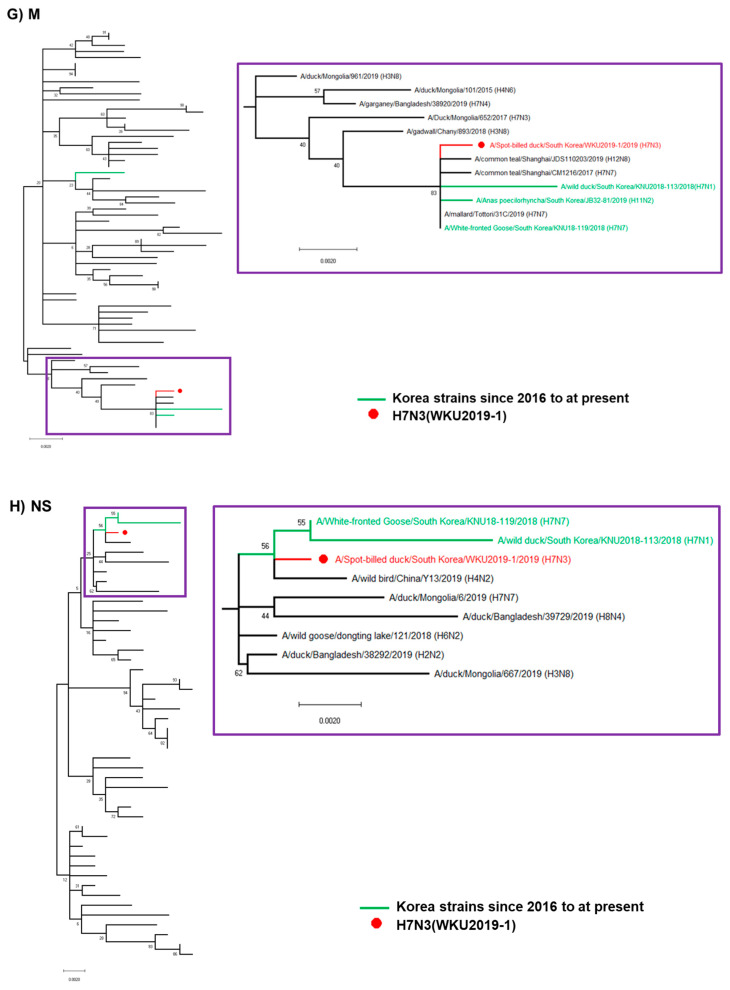
(**A**–**H**) Phylogenetic analysis of H7N3 (WKU2019-1) for eight gene segments. (**A**) PB2, (**B**) PB2, (**C**) PA, (**D**) HA, (**E**) NP, (**F**) NA, (**G**) M, (**H**) NS. (PB—polymerase basic protein; NP—nucleoprotein; HA—hemagglutinin; PA—polymerase acidic protein; NA—neuraminidase; M—matrix protein; NS—non-structural protein).

**Figure 2:**
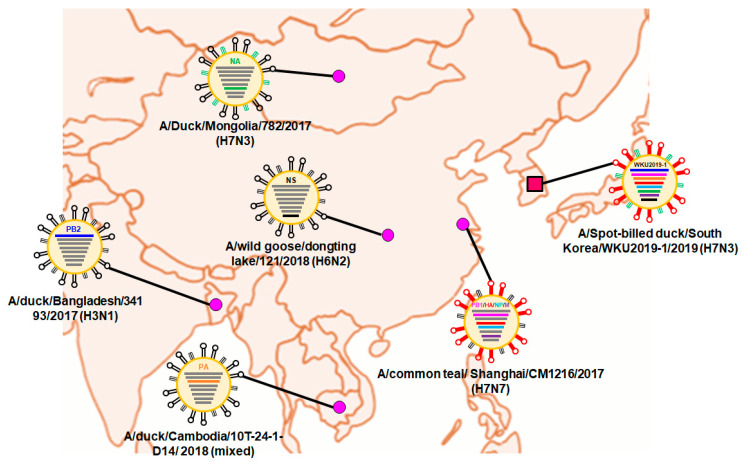
Locations of the putative origin of genomic compositions of the H7N3 (WKU2019-1).

Via evolutionary reassortment tracking analysis of our isolate from Figure 1, *NA* gene reassortment prevailed from the African region with links to Mongolian isolates in 2016. This was followed by the transmission of the *PB2* gene from South Korea through the H1N1 isolate to Bangladesh in 2016; then, the same gene reassortment occurred in H3N1, which was transmitted to China and rehabilitated with H7N8, before the *PB2* gene was finally reassorted to prevail in South Korea (Figure 2).

Similarly, the *NS* gene reassortment occurred in China (A/wild goose/dongting lake/121/2018 (H6N2)) before being transmitted to South Korea by wild ducks. Then, the same *NS* gene rehabilitated into H7N1 to H7N7 isolates. At present, the H7 subtype isolates are privileged in South Korea.

**Figure 3:**
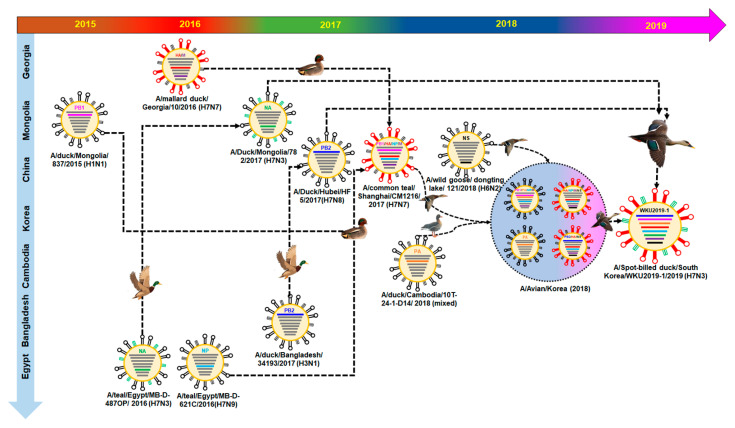
Original reassortment events of the novel avian influenza isolate H7N3 (WKU2019-1).

The backbone of the *HA* and *M* genes was due to the reassortment from Georgia isolates (H7N7) in 2016, likewise *PB1* and *NP* genes were formerly transmitted from Mongolia (H1N1) and Egypt (H7N9), respectively. Following that, four genes—*PB1*, *HA*, *M*, and *NP*—were collectively reassorted into A/common teal/Shanghai/CM1216/2017 (H7N7), which originated from China in 2017. Finally, the same gene was transmitted into South Korea from 2018 by various isolates of H7N1 and H7N1 through certain migratory birds like wild ducks and white-fronted geese. The detailed information on the evolutionary reassortment is presented in Figure 3.

International migration of the birds may have been involved in the transmission of the *PA* genes from the Cambodian isolate into South Korea from 2017–2018; our isolate may have been transmitted during this period. Additionally, there might have been a major H7N3 reassortment from China during the migration season of 2017–2018.

### 3.3. Molecular Characterization of the H7N3 (WKU2019-1) Isolate

The HA cleavage site of H7N3 (WKU2019-1) contained the PELPKGR↓GLF (↓denotes the cleavage site) sequence, with a monobasic amino acid arginine in the HA cleavage site, which is a marker for LPAIV H7 virus. Modification of HA receptor binding site (RBS) can lead a switch in the preference for glycans to bind preferentially to α-2,6-linked sialic acid receptors from avian to human, leading to an AIV pandemic [27]. We compared the HA protein of our isolate with four other isolated strains from birds: (1) a LPAIV isolate from a domestic duck from China (Zhejiang/2/2011 (H7N3) (designated as Zhejiang-2011 (H7N3)); (2) the backbone virus of our isolate, A/common teal/ Shanghai/CM1216/2017 (H7N7) (designated as Shanghai-2017 (H7N7)); (3) a reassortant HPAIV A/duck/Japan/AQ-HE30-1/2018 (H7N3) (designated as Japan-2018 (H7N3)) isolated from duck meat that was illegally taken onboard from China to Japan in 2018 containing the HA gene of Chinese H7N9 HPAIV; (4) and A/mallard/Korea/H836-10/2017 (designated as Korea-2017), which is closely related to H7N3 virus. Comparative analysis of HA RBS at position 138, 186, 190, 225, 226, 228 (H3 numbering) shows no mutation in amino acid residues (Table 2 and Figure S6). The monobasic residue at the HA cleavage site, with no notable mutation at the HA RBS, indicated that our H7N3 (WKU2019-1) isolate and reference strains, except for Japan-2011, to be LPAIV H7 viruses. However, the NA gene from our isolates contained isoleucine at position 223, indicating increased virulence in mammals [28,29]. Furthermore, internal genes were compared for any mutation that might have occurred and a summary of analysis is shown in Table 3. Several mutations were confirmed at the PB1, PB2, NP, NEP, and PA genes that are known to induce the polymerase and virulence activity in mammals. However, no mutation in NEP was discovered in any of our analyzed strains.

### 3.4. Growth Kinetics of H7N3 (WKU2019-1) Isolate in Mammalian Cell Culture

**Figure 4:**
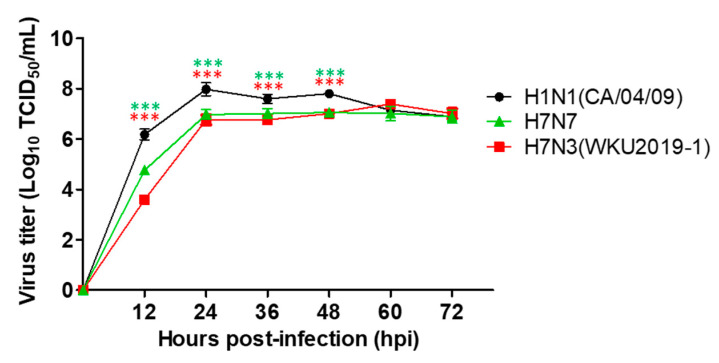
The virus growth kinetics of H7N3 (WKU2019-1) isolate in MDCK cells. MDCK cells were infected at an MOI of 0.01. The cell culture supernatant was harvested at different time-points (12, 24, 36, 48, 60, 72, and 84 h) after infection. The virus titer in cell culture supernatant was determined by an enzyme-linked immunosorbent assay (ELISA) using anti-influenza nucleoprotein to detect infected cells, and TCID_50_ was determined in MDCK cells. The data are represented as mean ± SD and calculated from three repeats, *** *p* < 0.001.

As the molecular characterization shows reassortment via mutation, our viral isolate could increase replication efficiency; therefore, in vitro examination of the viral replication of our isolate along with other isolates was performed. To evaluate the growth kinetics of our isolate, human-origin virus, A/California/07/2009 (H1N1), and H7N7, which is infectious to mammals, were used as controls. H1N1 replicated more efficiently in MDCK cells, as compared H7N3 (WKU2019-1) and A/mallard/Korea/KNUGPH12/2011 (H7N7) (Described as H7N7) (Figure 4). Raw data of TCID_50_ assay are shown in Figure S2.

### 3.5. Pathogenicity in Mice

**Figure 5:**
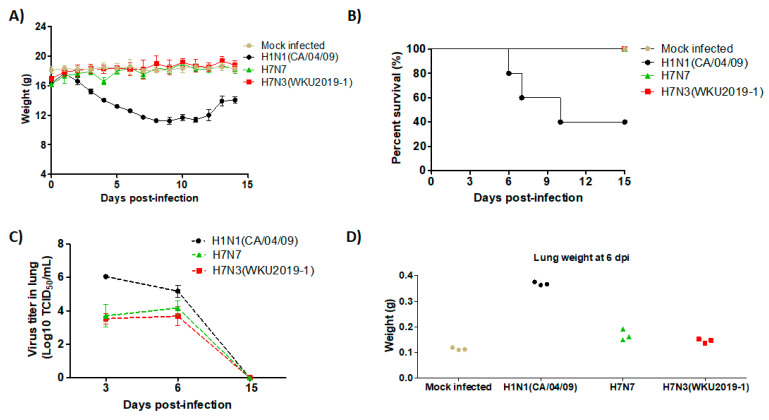
Pathogenicity of the H7N3 (WKU2019-1) isolate in vivo. For each virus strain, BALB/c mice were intranasally infected with 10^5^ EID_50_/mouse concentrations of the virus. (**A**) Mean body weight (*n* = 5), (**B**) the survival rates (*n* = 5), (**C**) virus titers in the lung (*n* = 3), (**D**) lung weight (*n* = 3) were noted.

**Figure 6:**
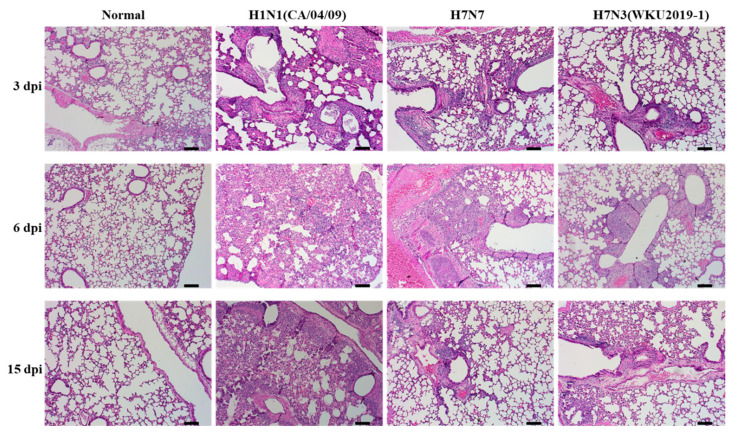
Histology of lung inflammation determined by hematoxylin and eosin (H&E) staining. For each isolate, BALB/c mice were intranasally infected with EID_50_ concentrations of the virus at 10^5^ EID_50_/mouse. The uninfected control (normal); H7N3 (WKU2019-1)-; H1N1 (CA/04/09)-; and H7N7-infected mouse lungs were collected and stained with H&E at days 3, 6, and 15 post-infection (dpi) (scale bar, 100 µm; original magnification × 100). Discussion.

We next examined the pathogenic potential of the new isolate by intranasally inoculating the virus in six-week-old female BALB/c mice. Mice infected with positive control, H1N1 virus, displayed severe clinical signs, including ruffled fur, depression, high body temperature, and gradual decrease in body weight from 3 dpi and gradually regained weight after 12 dpi (Figure 5A). In addition, three mice died on day 6, 7, and 10 post infection. However, our new isolate H7N3 (WKU2019-1) along with H7N7 did not display such clinical signs; all mice survived until the end of the experiment (Figure 5B), and a slight decrease in body weight at 3 dpi was observed. Virulence in lung after 3, 6, and 15 dpi were determined by TCID_50_ assay (Figure 5C). As shown in the results, control group H1N1 showed maximal virus titer in the lungs in comparison with H7N3 (WKU2019-1) and H7N7 at 3 dpi (6.05 ± 0.12, 3.53 ± 0.28; 3.71 ± 0.63 log_10_ (TCID_50_/mL), respectively) and these viral loads gradually decreased at 6 dpi (5.18 ± 0.32, 4.17 ± 0.39, 3.68 ± 0.53 log_10_ (TCID_50_/mL), respectively). In contrast, no virus was detected at 15 dpi in all groups, indicating that the virus was not yet well-adapted to murine infections. All raw data of the TCID50 assay are presented in Figure S3. In the histopathology study, for more confidence and comparison, all lung samples were submitted to the observatory staff after encoding as a blind test. The histopathological lesions were prominently observed in the lungs of the virus-challenged mice. Infected lungs at 3 and 6 dpi show a condensed penetration of neutrophils into the alveolar air spaces (Figure 6). Lungs weight and morphology of the H7N3 (WKU2019-1) and H7N7 virus infected mice were not significant different with normal mice as presented in Figure 5D, Figures S4 and S5.

LPAIVs of subtypes H7 and H5 have the ability to spontaneously mutate to HPAIV variants, causing high mortality. The first outbreak of HPAIV caused by subtype H7N3 appeared in Pakistan in 1995 and affected 3.2 million birds, primarily breeders and boilers. Since then, it has caused outbreak in Chile (2004) [63], the Netherlands (2003) [64], British Colombia (2004) [65], and Mexico (2012) [66], causing heavy losses in the poultry industry. Although Kim et al. reported the first isolations of two H7N3 strains during their 2008–2011 systemic surveillance program [13] in the Korean peninsula, detailed molecular and pathological studies from South Korea are scarce.

During our influenza surveillance, the H7N3 strain was isolated, and through further studies was subtyped as an LPAIV isolate with a HA cleavage site sequence PELPKGR↓GLF. According to phylogenetic analysis, the HA, PA, and M genes were closely related to A/wild duck/South Korea/KNU18-114/2018 (H7N7), while the genes of PB1, NS, and NP were closer to the Korean isolates—A/wild fronted Goose/South Korea/KNU18-119/2018 (H7N7) and A/wild duck/South Korea/KNU18-114/2018 (H7N7); the genes of PB2 was similar to that of china isolate, A/ duck/Hubei/HF5/2017 (H7N8); and that of NA was similar to that of Mongolian isolate A/duck/Mongolia/782/2017 (H7N3).

In the H7 subtype, most of the residues, including 138 and 221, were highly conserved. The single substitutions at those two sites (S138A or T221P) does not cause obvious changes in the receptor-binding properties and retained avian receptor-binding specificity unless G186V substitution was displayed. Further negligible binding to the human RBS was observed, even if the three other sites were replaced by hydrophobic residues (S138A, T221P, and Q226L). V186 is the key determinant of avian-specific H7N9 HA obtaining human receptor-binding capacity [67]. However, no such mutation was observed with our H7N3 (WKU2019-1) isolate as compared to the HPAIV strain Japan-2018 (H7N3) (Table 2). This indicates G186V to be a key determinant of HPAIV H7N3. Similarly, the deletion of variable length (from 50–70 amino acids) in the NA stalk is a molecular feature frequently found to represent an adaptation of wild bird viruses to poultry. However, such a deletion was absent in our isolate as well as in the HPAIV Japan-2018 (H7N3) reference strain. Nevertheless, several substitutions have been identified in the genes of PB2, NP, M1, NA (Table 3), which were previously assigned to enhance polymerase activity that caused the virus to replicate dramatically and enhance virulence in mice. The result was supported by in vitro experiments as demonstrated by the kinetic growth dynamics of each virus shown in Figure 3. The H7N3 (WKU2019-1) virus replicated rapidly in the mammalian MDCK cells, and replication were stabilized at 24 hpi. Similarly, the new isolate exhibited moderate pathogenicity in mice, with noticeable virus titers in the lung 3 and 6 dpi compared to the control, which was infected with human-origin H1N1. This result corroborates with the results of the molecular characterization. However, the Shanghai-2017 (H7N7) backbone of our WKU-2019-1 H7N3 virus showed that with a 10^6^ EID_50_ virus inoculation, the highest virus titer was achieved at 3 dpi with drastic weight loss and severe diffused pneumonia. The difference in the two experiments was the concentration of virus used, which might be the reason for such a difference in the virus titer achieved and the histopathological changes in lungs. The H7N3 (WKU2019-1) isolate carries mutations in the proteins of PB2 (H447Q) [33], PB1 (K328N, T/D/V/R/A127N), and PA (F227S) [55], which lead to increased virulence and adaptation to mammals. However, none of the isolates that were compared for the mutational analysis carried the human-adaptation marker, E627K, in the PB2 gene. Our WKU-19 H7N3 isolate still bear the avian host-specific residue possessing the Q226L in HA. In the 1918 influenza virus mutation in PA, E382D was observed to be responsible for human adaptation [38]. Furthermore, the PA protein of the backbone virus, Shanghai-2017 (H7N7), and Japan-2018 (H7N3) isolate shows an E328D mutation. However, no such mutation was observed with our H7N3 (WKU2019-1) isolate. In this study, an absence of key amino acid mutations in our isolate show increased adaptation of AIVs to humans; however, mutations at several genes were observed to increase virulence in mammals and mice. A 2013 study from Bangladesh reported that a H9N2 AVI isolated from chickens/quails from a live market carried three internal genes—NS, PA, and PB1—from a HPAIV subtype H7N3 from Pakistan. The H9N2 virus replicated well without any clinical signs and symptoms, and spread via direct contact [45]. A recent study provided evidence for LPAIV-to-HPAIV mutation during a H7N3 infection in a turkey farm in the Netherlands [65]. Although H7N3 (WKU2019-1) is a LPAIV isolate, to mitigate the risk for human infection and the potential for genetic reassortment, its movement should to be monitored frequently.

In conclusion, the ultimate goals and objectives of our surveillance was to detect Avian influenza rather than any other virus and microorganism. Furthermore, RNA isolated from initial feces failed to read the virus by NGS hence virus amplified from allatonic fluid was used for sequencing and subtyping which was the limitation of our study. However, this study is first to broaden the knowledge of Korean H7N3 isolates from evolution to mammalian cell expressions and in vivo characterization. Continuous monitoring and molecular characterization of the H7N3 virus will be required for the broader understanding of evolutionary dynamics of the virus; further improving control measures is recommended.

## Figures and Tables

**Table 1:** Virus strains from GenBank database with highest nucleotide identities when compared with the H7N3 (WKU2019-1) isolate in this study.

Gene	Gene Bank ID	Reference Strain Accession ID	Origin	Per Ident (%)
*PB2*	MT845654	KY402062	A/hooded crane/Korea/1176/2016 (H1N1)	99.25 (2280/2280)
		MH458919	A/Duck/Hubei/HF5/2017 (H7N8)	99.17 (2280/2280)
		MH791830	A/duck/Bangladesh/34193/2017 (H3N1)	99.17 (2295/2280)
*PB1*	MT845655	MN602508	A/White-fronted Goose/South Korea/KNU18-119/2018 (H7N7)	99.61 (2287/2341)
		MN602505	A/wild duck/South Korea/KNU18-114/2018 (H7N7)	99.56 (2287/2341)
		MK554565	A/common teal/Shanghai/CM1216/2017 (H7N7)	99.56 (2252/2341)
*PA*	MT845656	MN602506	A/wild duck/South Korea/KNU18-114/2018 (H7N7)	99.54 (2180/2223)
		EPI_ISL_309223	A/mallard/Korea/H836-10/2017 (H7N3)	99.45 (2200/2223)
		MN703036	A/duck/Cambodia/10T-24-1-D14/2018 (mixed)	99.36 (2209/2223)
*HA*	MT845657	MN483232	A/wild duck/South Korea/KNU18-114/2018 (H7N7)	99.82 (1683/1731)
		MN483237	A/White-fronted Goose/South Korea/KNU18-119/2018 (H7N7)	99.64 (1683/1731)
		MN480525	A/wild duck/South Korea/KNU2018-113/2018 (H7N1)	99.53 (1708/1731)
		MK554567	A/common teal/Shanghai/CM1216/2017 (H7N7)	99.51 (1639/1731)
*NP*	MT845658	MN208011	A/teal/Egypt/MB-D-487OP/2016 (H7N3)	99.66 (1563/1497)
		MN480533	A/wild duck/South Korea/KNU2018-113/2018 (H7N1)	99.53 (1541/1497)
		MK554568	A/common teal/Shanghai/CM1216/2017 (H7N7)	99.53 (1499/1497)
*NA*	MT845659	EPI_ISL_327473	A/Duck/Mongolia/782/2017 (H7N3)	97.85 (1446/1455)
		MN208013	A/teal/Egypt/MB-D-487OP/2016 (H7N3)	99.48 (1450/1455)
		MN208042	A/northern shoveler/Egypt/MB-D-690C/2016 (H7N3)	98.34 (1450/1455)
*M2, M1*	MT845660	MN483235	A/wild duck/South Korea/KNU18-114/2018 (H7N7)	100 (982/1028)
		MN584917	A/wild duck/South Korea/KNU2018-113/2018 (H7N1)	99.90 (1006/1028)
		MK554570	A/common teal/Shanghai/CM1216/2017 (H7N7)	99.90 (990/1028)
*NEP, NS1*	MT845661	MN483241	A/White-fronted Goose/South Korea/KNU18-119/2018 (H7N7)	99.77 (866/873)
		MH727484	A/wild goose/dongting lake/121/2018 (H6N2)	99.65 (864/873)
		MN480542	A/wild duck/South Korea/KNU2018-113/2018 (H7N1)	99.19 (865/873)

**Table 2:** Comparison of the hemagglutinin (HA) receptor-binding sites and neuraminidase (NA) gene segments of the novel avian H7N3 isolate and those of high and low pathogenicity avian H7N3 isolates. (“-”—no amino acid were found).

Virus Strain	HA Receptor-Binding Residues (H3 Numbering)											NA					
	Cleavage Sites	138	158	183	186	190	221	225	226	228	391	Deleted Range from 50–70	26	106	223	373	394
WKU2019-1 (H7N3)	PELPKGR↓GLF	A	T	H	G	E	P	G	Q	G	Q	No deletion	V	I	I	F	Q
Zhejiang-2011 (H7N3)	PEIPKGR↓GLF	A	T	H	G	E	P	G	Q	G	N	No deletion	I	I	I	F	Q
Japan-2018 (H7N3)	PEVPKRR↓TAR	A	T	H	V	E	P	G	Q	G	Q	No deletion	I	I	I	F	Q
Korea-2017	PELPKGR↓GLF	A	T	H	G	E	P	G	Q	G	N	No deletion	I	I	I	F	Q
Zhejiang-2011 (H7N7)	PELPKGR↓GLF	A	T	H	G	E	P	G	Q	G	Q	-	-	-	-	-	-

**Table 3:** Summary of data obtained from the mutational analysis of eight genes from AIVs of multiple avian species with the H7N3 (WKU2019-1) isolate. (“-”—no amino acid were found).

Viral Protein	Amino Acid	H7N3 (WKU2019-1)	Zhejiang-2011 (H7N3)	Japan-2018 (H7N3)	Korea-2017 (H7N3)	Zhejiang-2011 (H7N7)	Phenotype	References
PB2	T63I	I	I	I	I	I	Pathogenic in mice	[30]
	L89V	V	E	V	V	V	Enhanced polymerase activity, Increased virulence in mice	[31]
	K251R	R	R	R	R	R	Increased virulence in mice	[32]
	G309D	D	D	D	D	D	Enhanced polymerase activity, Increased virulence in mice	[31]
	Q368R	R	R	R	R	R	Increased polymerase activity, Increased virulence in mammals	[33,34]
	H447Q	Q	Q	Q	Q	Q	Increased polymerase activity, Increased virulence in mammals	
	I471T	T	T	T	T	T	Change the surface electrostatic potential drastically	[35]
	R477G	G	G	G	G	G	Enhanced polymerase activity, Increased virulence in mice	[31]
	I495V	V	V	V	V	V	Enhanced polymerase activity, Increased virulence in mice	
	E627K	E	E	E	E	E	Human adaptation marker	
	A676T	T	T	T	T	T	Enhanced polymerase activity, Increased virulence in mice	[31]
PB1	D/A3V	V	V	V	V	-	Increased polymerase activity, Increased virulence in mammals	[33,34]
	L13P	P	P	P	P	P	Increased polymerase activity, Increased virulence in mammals, Mammalian host marker, Amantadine resistance	[35,36]
	R207K	K	K	K	K	K	Increased polymerase activity in mammalian cells	[37]
	K328N	N	N	N	N	N	Increased polymerase activity, Increased virulence in mammals	[33,34]
	S375N	N	N	N	N	N	Increased polymerase activity, Increased virulence in mammals, Human host marker	[33,34,38]
	H436Y	Y	Y	Y	Y	Y	Increased polymerase activity and virulence in mallards, ferrets and mice	[39]
	A469T	T	T	T	T	T	Conferred in contact transmissibility in guinea pigs.	[40]
	L473V	V	V	V	V	V	Increased polymerase activity and replication efficiency	[41]
	V652A	A	A	A	A	A	Increased virulence in mice	[32]
	M677T	T	T	T	T	T	Pathogenic in mice	[30]
PB1-F2	N66S	S	N	N	N	S	Increased virulence in mammals	[42,43,44]
	T68I	T	T	-	T	T	Increased virulence in mammals	[45]
NEP	A/P42S	S	S	A	S	-	Increased virulence in mammals, Antagonism of interferon induction	[46]
	T/D/V/R/A127N	N	N	R	N	-	Increased virulence in mammals	[46,47]
	V149A	A	A	A	A	-	Pathogenicity in mice, Antagonism of interferon induction	[48]
	T47A	E	E	E	E	E		
	T48N	A	A	S	A	A		
	M51I	R	R	R	R	R		
NP	V41I	I	I	I	I	I	Might contribute to viral transmissibility	[49]
	I109V	V	I	I	I	I	Mammalian host specific mutation	[45]
	R214K	R	N	R	R	R	Mammalian host specific mutation	
	F313Y	F	A	F	F	F	Mammalian host specific mutation	
	E372D	T	S	T	T	T	Mammalian host specific mutation	
	V105M	V	M	M	M	V	Contribute to the increased virulence of the H9N2	[50]
	D210E	E	E	E	E	E	Might contribute to viral transmissibility	[49]
	F253I	I	A	I	I	I	Results in attenuated pathogenicity of the virus in mice	[51]
	I353V	V	V	I	I	V	Increased virulence in mice	[32]
PA	D3V	D	E	E	E	-	Contribute to the increased virulence of the H9N2	[50]
	S37A	A	A	A	A	A	Significantly increased viral growth and polymerase activity in mammalian cells	[52]
	V44I	V	V	V	V	V	Enhance the replicative ability of an H5N1 virus in A549 cells and enhance its pathogenicity in mice	[53]
	H266R	R	R	R	R	R	Increased polymerase activity, Increased virulence in mammals and birds	[54]
	F277S	S	S	S	S	S	Adapt to mammalian hosts	[55]
	C278Q	Q	Q	Q	Q	Q	Adapt to mammalian hosts	
	E382D	E	E	D	E	D	Human host marker	[38,56]
	N383D	D	D	D	D	D	Enhanced the pathogenicity and viral replication of H5N1 virus in mice	[57,58]
	S/A515T	T	T	T	T	T	Increased polymerase activity, Increased virulence in mammals and birds	[54]
	L653P	P	P	P	P	P	Adapt to mammalian hosts	[55]
PA-X	R195K	R		K			Increased virulence in mammals	[28]
M1	V15I	V	I	S	I	V	Increase pathogenicity to mice	[59]
	V15I/T	V	S	V	I	V	Increased virulence in mammals	[28,29,60]
	N30D	D	F	D	D	D	Increased virulence in mammals	[61]
	V115I	V	V	V	L	-	Human host marker	[62]
	T121A	T	T	T	A	-	Human host marker	[61]
	M128R	M	M	M	L	-	Increased virulence in mice	[37]
	A166V	V	V	A	V	-	Contribute to the increased virulence of the H9N2.	[50]
	S183A	S	S	S	T	-	Resulted in the failure of virus production	[62]
	T185A	T	T	T	K	-	Resulted in the failure of virus production	
	T215A	A	A	A	R	-	Increased virulence in mammals	
M2	L55F	L	L	F	Y	L	Enhanced Transmission	[56]
	L26F	L	L	L	I	L	Adamantine resistance mutation	[45]
	S31N	S	S	N	I	S	Adamantine resistance mutation/Antiviral resistance S31 (amanta)	[59]

## Data Availability

The data presented in this study are available in this article and Supplementary Materials.

## Supplementary Materials

The following are available online at https://www.mdpi.com/article/10.3390/v13050856/s1, Figure S1: Amplification of HA and NA genes by influenza universal primer (1–5 Selected colonies; * Target band). Figure S2: Raw ELISA data of TCID50 assay for the detection of (A) H7N3 (WKU2019-1), (B) H1N1 (CA/04/09), and (C) H7N7 growth kinetics in MDCK cells. Figure S3: Raw ELISA data of TCID50 assay for viral load shedding in lungs after (A) 3, (B) 6, and (C) 15 days’ post-infection. Figure S4: Lungs from normal and infected mice at 3, 6, and 15 days post-infection. Scale bar: 0.5 cm. Figure S5: Lung weight at days (A) 3 and (B) 15 post-infection. Figure S6: Partial aliment of HA gene segment. Table S1: Detailed NGS analysis information of H7N3 (WKU2019-1) isolated from feces samples. Table S2: Detailed NGS analysis information of H7N3 (WKU2019-1) isolated from allatonic fluid.
