# Supplementary material for: Genetic Characterization and Pathogenesis of Avian Influenza Virus H7N3 Isolated from Spot-Billed Ducks in South Korea, Early 2019
Source: Viruses. 2021 May 7;13(5):856. doi: 10.3390/v13050856 (PMC8151380; doi:10.3390/v13050856)
Supplement: Supplementary file 1 [file viruses-13-00856-s001.zip › viruses-1171054-supplementary.pdf]

Supplementary data

Genetic Characterization and Pathogenesis of Avian Influenza Virus H7N3 Isolated from Spot-Billed Ducks in South Korea, Early 2019

Thuy-Tien Thi Trinh [1,†], Indira Tiwari [1,†], Kaliannan Durairaj [1,†], Bao Tuan Duong [1], Anh Thi Viet Nguyen [1], Hien Thi Tuong [1], Vui Thi Hoang [1], Duong Duc Than [1], SunJeong Nam [2], Seon-Ju Yeo [3,*] and Hyun Park [1,*]

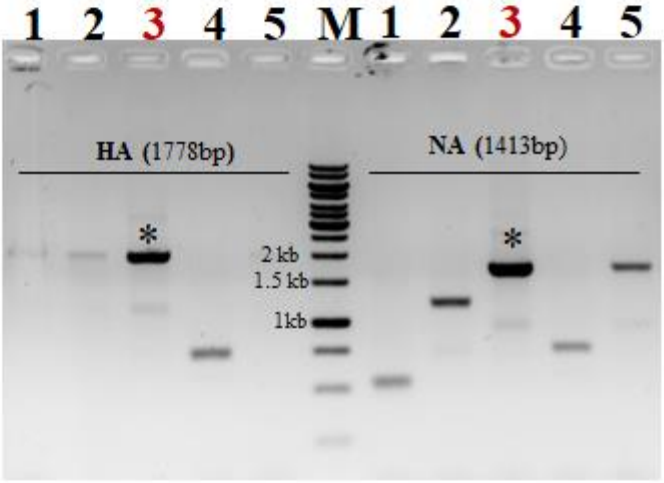

Figure S1. Amplification of HA and NA gene by influenza universal primer. (1–5; Selected colonies, *; Target band).

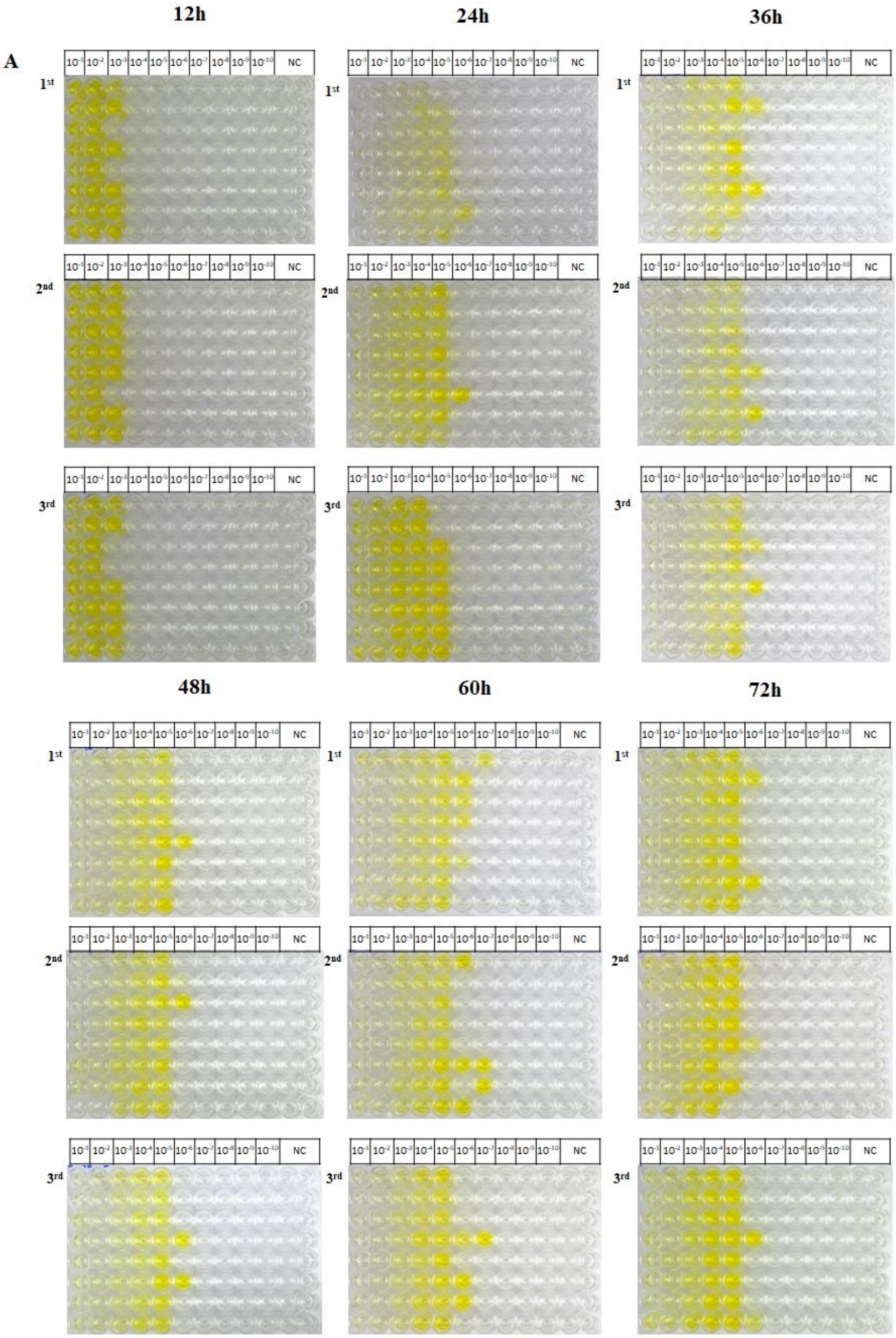
A
12h
24h
36h
48h
60h
72h
1st
2nd
3rd
10-1 10-2 10-3 10-4 10-5 10-6 10-7 10-8 10-9 10-10 NC

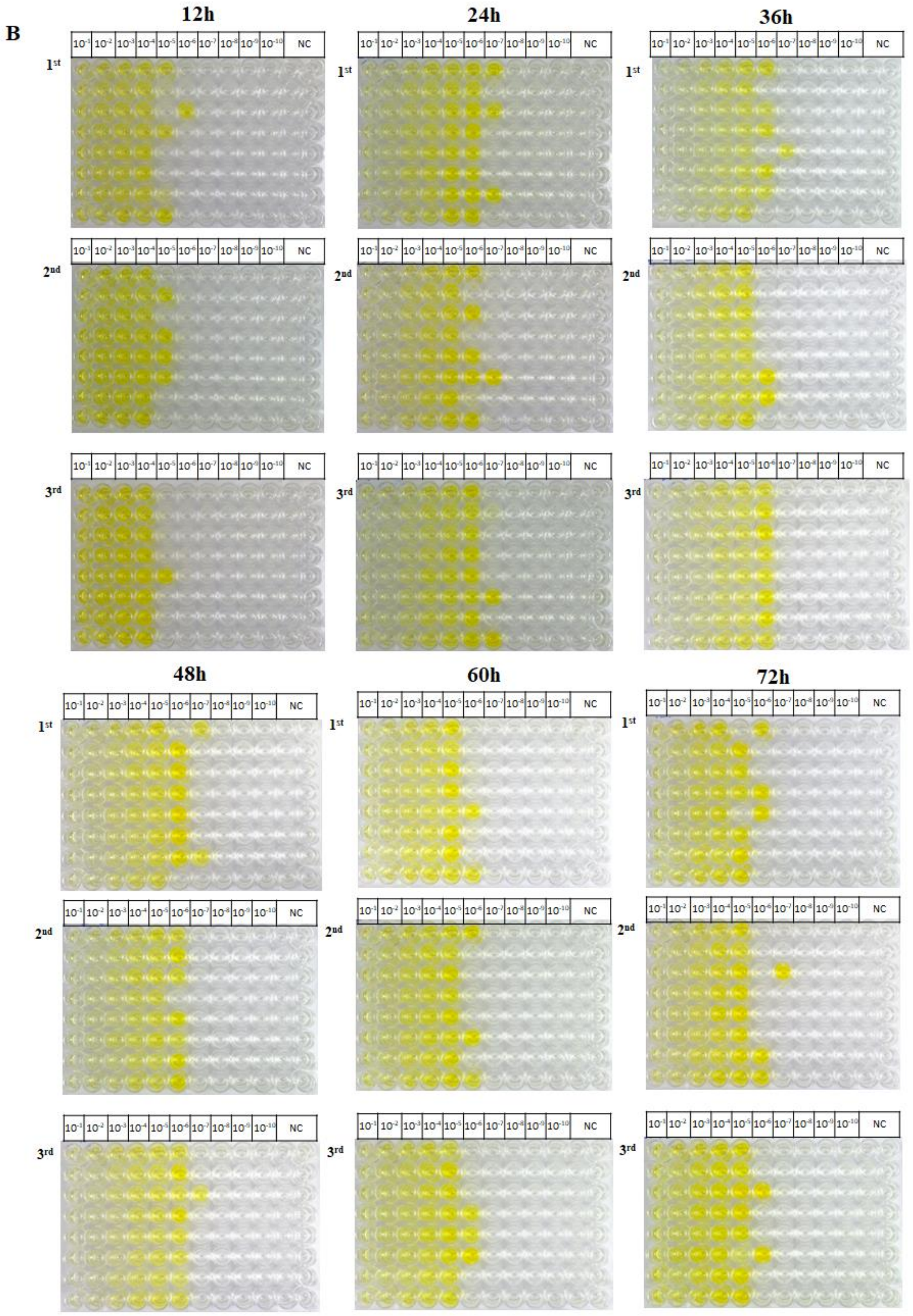
B
12h
24h
36h
48h
60h
72h
1st
2nd
3rd
10-1 10-2 10-3 10-4 10-5 10-6 10-7 10-8 10-9 10-10 NC

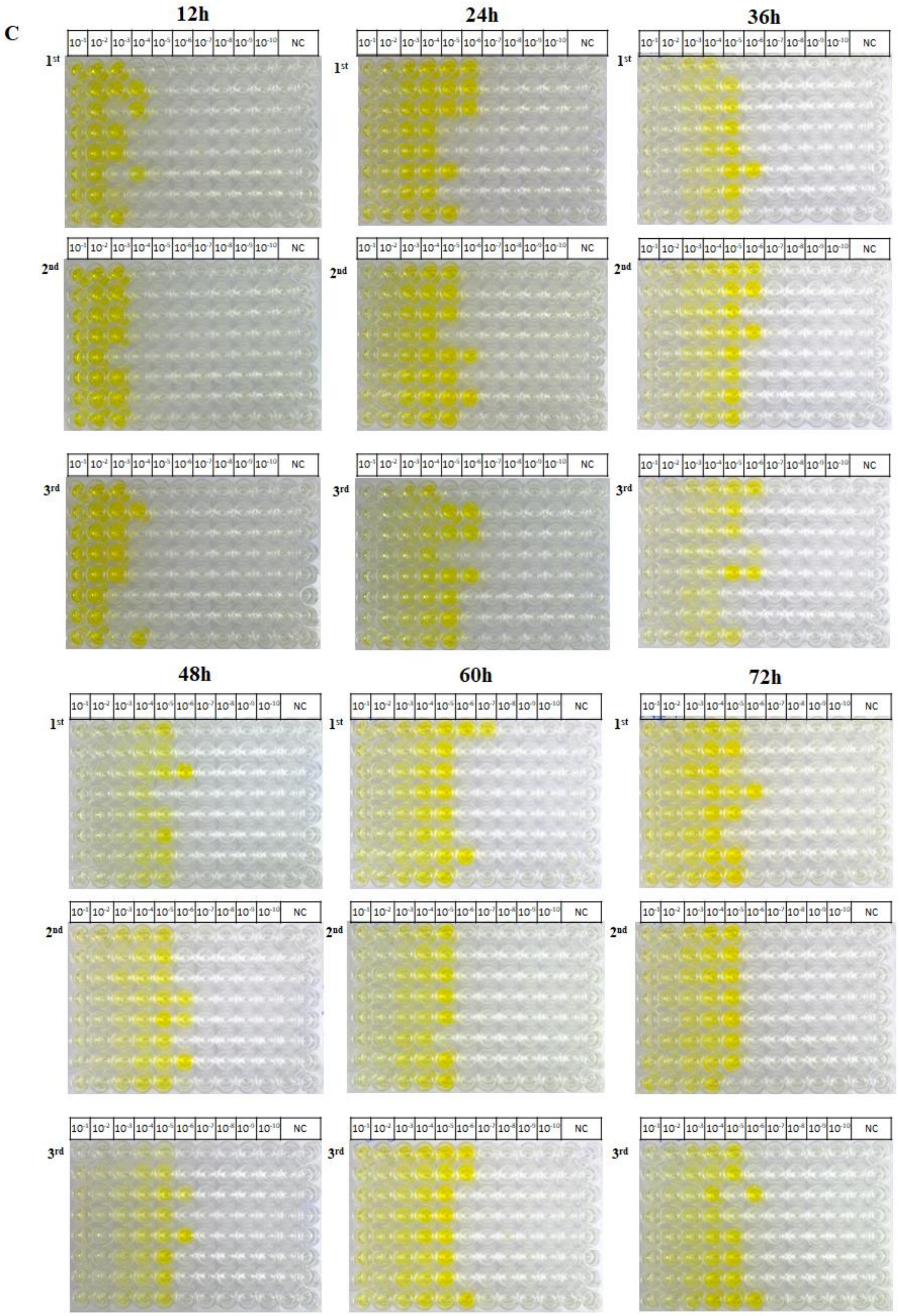

Figure S2. Raw ELISA data of $TCID_{50}$ assay for the detection of (A) H7N3 (WKU2019-1), (B) H1N1 (CA/04/09), and (C) H7N7 growth kinetics in MDCK cells.

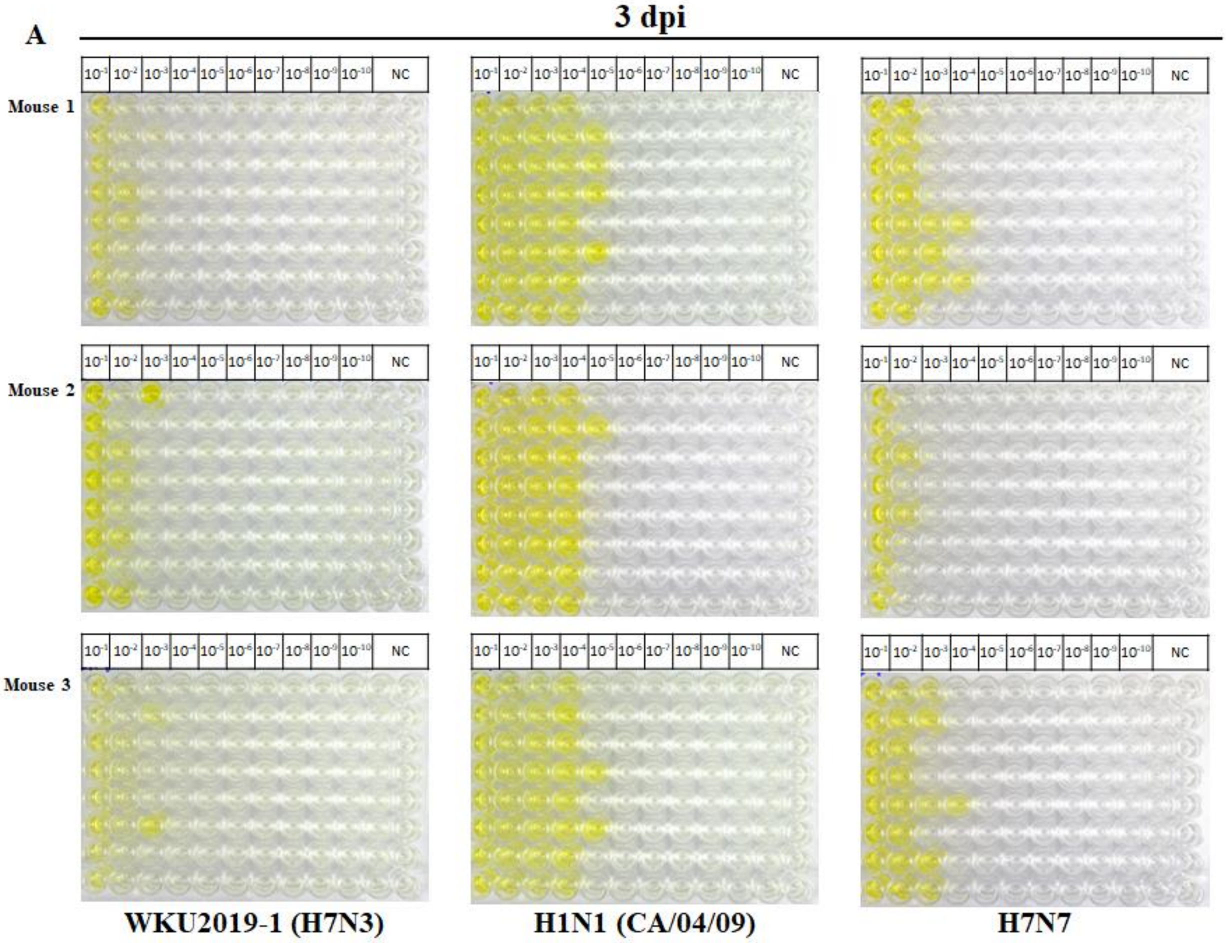
A
3 dpi
Mouse 1
Mouse 2
Mouse 3
10⁻¹ 10⁻² 10⁻³ 10⁻⁴ 10⁻⁵ 10⁻⁶ 10⁻⁷ 10⁻⁸ 10⁻⁹ 10⁻¹⁰ NC
WKU2019-1 (H7N3)
H1N1 (CA/04/09)
H7N7

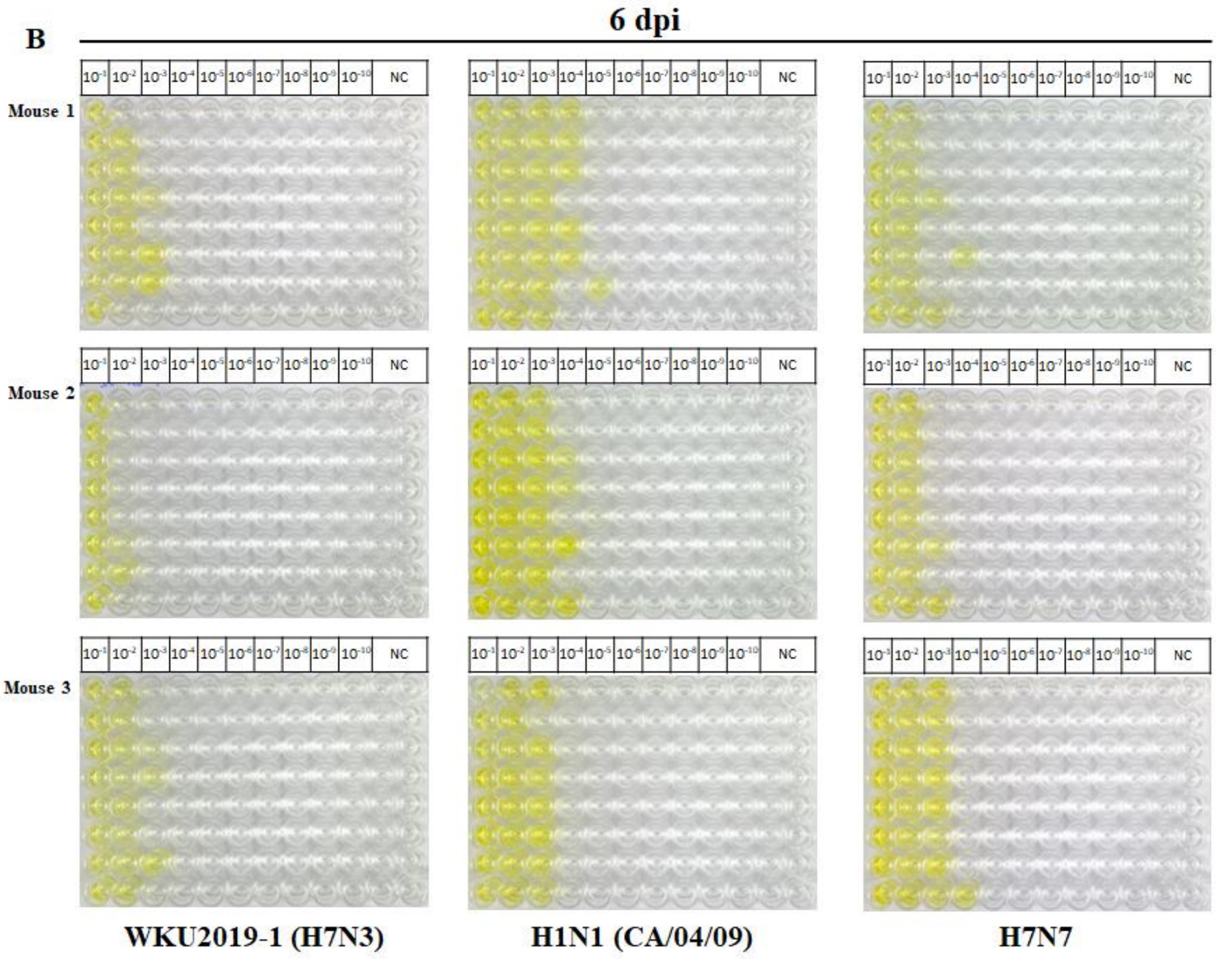
B
6 dpi
Mouse 1
Mouse 2
Mouse 3
NC
WKU2019-1 (H7N3)
H1N1 (CA/04/09)
H7N7

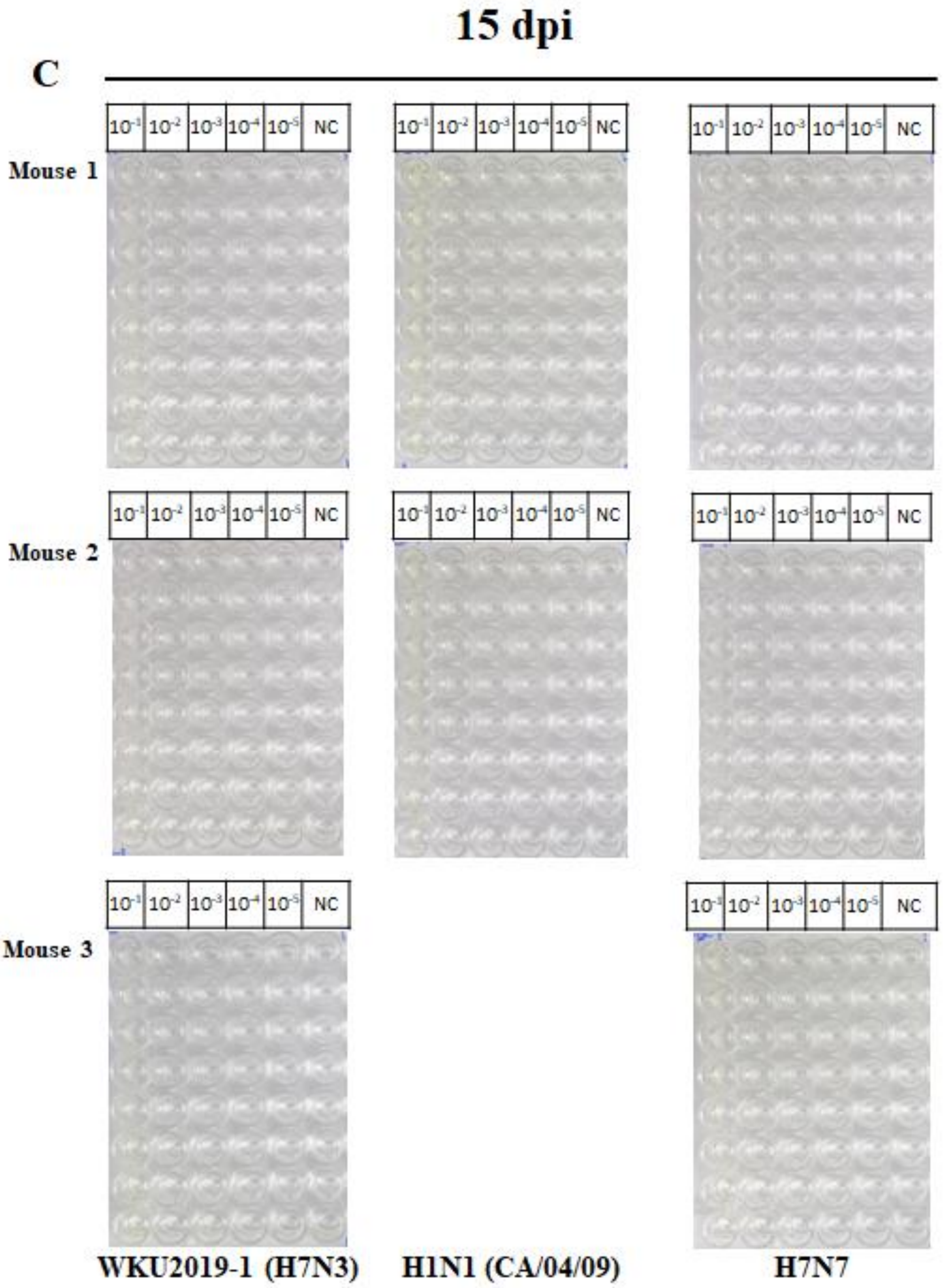

Figure S3. Raw ELISA data of $TCID_{50}$ assay for viral load shedding in lungs after (A) 3, (B) 6, and (C) 15 days post-infection.

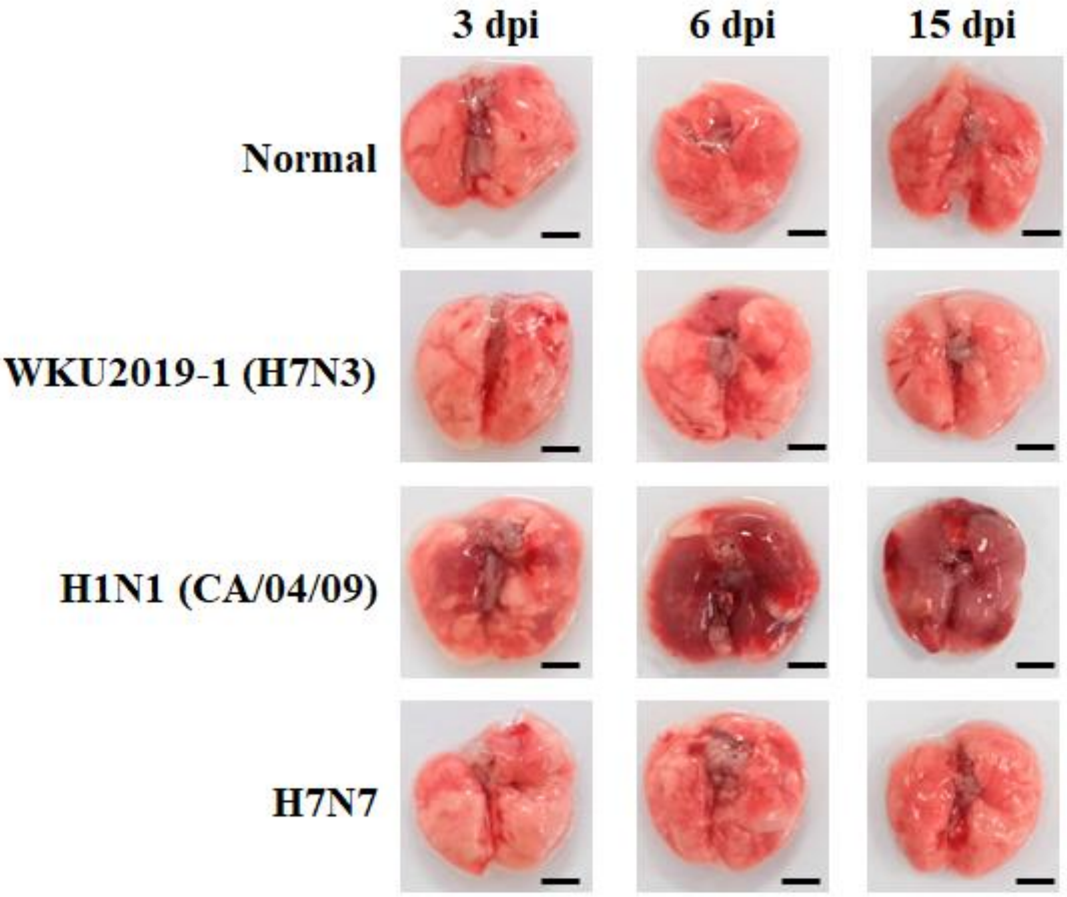

Figure S4. Lungs from normal and infected mice at 3, 6, and 15 days post-infection. Scale bar: 0.5 cm.

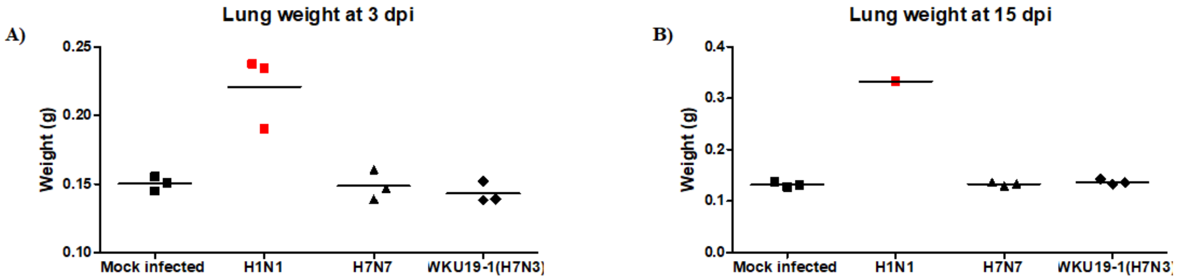

Figure S5. Lung weight at days (A) 3 and (B) 15 post-infection.

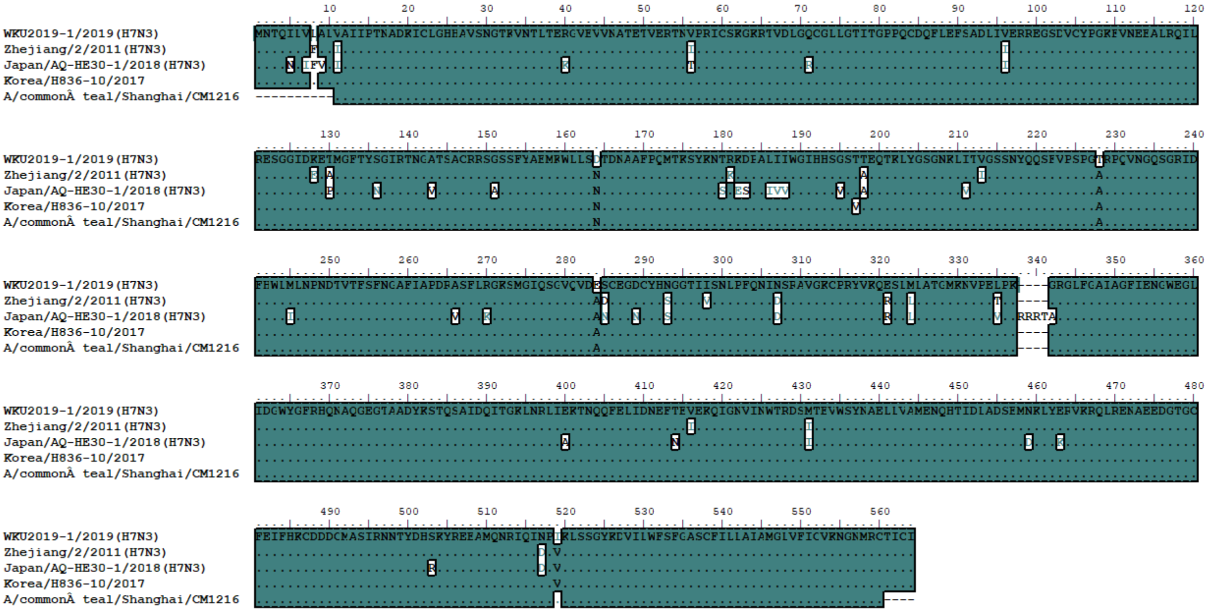

Figure S6. Partial aliment of HA gene segment.

Table S1. Detailed NGS analysis information of H7N3 (WKU2019-1) isolated from feces samples.

Gene	Sample Information								ORF Analysis		
	# of Pre-processed reads	# of Influenza Virus extracted reads	# of non-Influenza Virus reads	Virus reads %	#M_Reads	Unique Matches	S_Con._bp	%Cov.(S/R)	Length	S_position	E_position
PB2					1	0.90%	-	4.22%	2,304	2	2,305
PB1					6	5.50%	-	11.76%	2,274	23	2,296
PA					1	0.90%	-	2.42%	2,157	19	2,175
HA	14,874,694	110	14,874,584	0.00%	5	4.50%	-	9.59%	1,818	3	1,820
NP					3	2.70%	-	8.93%	1,542	2	1,543
NA							-		1,437	2	1,438
M2, M1					19	17.30%	-	44.17%	759	3	761
NEP, NS1							-		693	1	693

Table S2. Detailed NGS analysis information of H7N3 (WKU2019-1) isolated from allantoic fluid.

Gene	Gene Bank ID	Reference Information Used for Mapping	Sample Information	ORF Analysis (Blast P, rank=1)	
				Matched Reference	results

		Reference name	Ref. ID	Ref. length	# of Pre-processed reads	# virus reads	% virus reads	#Mapped reads	Total Mapping avg. depth	%Cov.	Length	ID	Length	%Identity
PB2	MT845654	A/mallard/Bavaria/185-26/2008(H1N1)	HQ259229	2,306				12,200			2,307	AXK58929.1	759	756/759 (99%)
PB1	MT845655	A/mallard duck/Georgia/10/2016(H7N7)	MF694021	2,341				58,963			2,341	BAJ83375.1	757	751/757 (99%)
PA	MT845656	A/mallard/Bavaria/185-26/2008(H1N1)	HQ259231	2,221				20,815			2,223	AXK58968.1	716	712/716 (99%)
HA	MT845657	A/mallard duck/Georgia/10/2016(H7N7)	MF694244	1,732	8,900,256	7,189,378	80.78	68,166	7,841	100 %	1,731	QEQ76131.1	560	558/560 (99%)
NP	MT845658	A/aquatic bird/South Korea/sw001/2015(H7N1)	MF987896	1,497				34,473			1,497	ADP07574.1	498	495/498 (99%)
NA	MT845659	A/tufted duck/Georgia/1/2012(H2N3)	MF147102	1,453				47,446			1,455	BAU50751.1	469	465/469 (99%)
M2, M1	MT845660	A/mallard duck/Georgia/10/2016(H7N7)	MF694150	1,027				258,104			1,028	Q08IG8.1	252	252/252 (100%)
NEP, NS1	MT845661	A/mallard/Bavaria/185-26/2008(H1N1)	HQ259236	873				102,240			873	QHG62516.1	230	229/230 (99%)
